# A lifeline on wheels: perspectives of stakeholders on the implementation and impact of a mobile medications for opioid use disorder unit

**DOI:** 10.1186/s13722-026-00664-4

**Published:** 2026-04-11

**Authors:** Augustine Kang, Amelia Bailey, Linda Hurley, Rosemarie Martin

**Affiliations:** 1 Boston Children’s Hospital, 300 Longwood Ave, Boston, MA 02115 USA; 2 Brown University School of Public Health, Providence, RI USA; 3 CODAC Behavioral Healthcare Inc, Providence, RI USA; 4 University of Massachusetts Chan Medical School, Worcester, MA USA

**Keywords:** Mobile health units, opioid use disorder, medications for opioid use disorder, treatment access, harm reduction

## Abstract

**Introduction:**

Mobile health units (MHUs) providing medications for opioid use disorder (MOUD) have emerged as a critical strategy to address gaps in opioid treatment access, particularly for marginalized populations. Research has yet to explore stakeholder perspectives on MHUs’ implementation, challenges, and long-term sustainability.

**Methods:**

We conducted semi-structured interviews with 15 stakeholders, including MHU staff, administrators, community partners, and policymakers. Interviews explored experiences with the MHU, barriers and facilitators to patient engagement, and operational challenges. Data were transcribed, coded, and analyzed using a template thematic approach to identify key themes related to implementation and sustainability.

**Results:**

Stakeholders endorsed the MHU as a highly accessible and flexible intervention that reduces barriers for people experiencing homelessness, economic instability, and transportation limitations. The MHU facilitated strong patient-provider relationships and access to harm reduction, primary care, and social services. Challenges included staffing shortages, inconsistent funding, limited clinical space, and environmental barriers. Additionally, stigma, political resistance, and law enforcement interactions affected service delivery and patient engagement.

**Discussion:**

Findings highlight the importance of policy and funding mechanisms to ensure the long-term viability of MHUs. Stakeholders recommended expanding outreach, increasing staffing, and integrating additional services. Addressing these challenges is essential to sustaining MHUs as an effective public health intervention for opioid use disorder.

## Introduction

The use of mobile health units (MHUs) to deliver medications for opioid use disorder (MOUD) has emerged as a key strategy for increasing treatment access among individuals with opioid use disorder (OUD). Initially approved by the U.S. Drug Enforcement Administration in 1988, MHUs were designed to reach underserved populations in both urban and rural areas, providing methadone-based treatment in locations with limited opioid treatment programs (OTPs) [1]. Early models of the MHU demonstrated relative success, with mobile methadone clinics increasing engagement among individuals with substance use disorders [2]. However, in 2007, the Drug Enforcement Administration placed a moratorium on new methadone-providing MHUs, significantly restricting their expansion until the policy was reversed in 2021 [1]. Since then, the number of approved methadone MHUs in the U.S. has risen dramatically, from 8 in 2022 to 64 by early 2025 [3]. Regardless, a recent review identified that MHUs that deliver MOUD are under-utilized, indicating both a need and an opportunity to expand these services [4]. 

MHUs reduce barriers to care by eliminating common logistical obstacles such as transportation difficulties, long wait times, and restrictive program policies [5]. Unlike brick-and-mortar OTPs, which often require frequent in-person visits, mobile units improve treatment retention by potentially offering more flexible access to MOUD [4, 5]. In addition to delivering MOUD, mobile units frequently serve as comprehensive health hubs, offering primary care, harm reduction services, and case management support [6]. 

Compared to brick-and-mortar OTPs, MHUs disproportionately serve marginalized populations, including unhoused individuals, racialized individuals, and uninsured patients [7]. Of note, about 13% of adults initiating OUD treatment are unhoused [8]. These units are particularly valuable in regions with high overdose rates and inadequate access to MOUD services, such as rural communities and areas with few existing OTPs [9]. While MHUs have demonstrated effectiveness in engaging high-risk populations, research on the process of MHU implementation, particularly in settings where multiple forms of MOUD (methadone, buprenorphine, and naltrexone) are available [4]. 

In response to the Drug Enforcement Administration’s revised policy, CODAC Behavioral Healthcare, Rhode Island’s largest OTP provider, launched an MHU in July 2022 to serve areas with high overdose rates and limited treatment access. The unit operates in two primary roles: (1) As an OTP – Prescribing and dispensing MOUD six days a week in Woonsocket, a city with one of the highest per capita overdose rates in Rhode Island [10], and (2) As a community outreach service – Providing harm reduction, counseling, and linkages to care in under-resourced areas. These MHUs are staffed by a licensed clinical provider and supporting administrative staff. CODAC’s MHU offers a unique opportunity to evaluate the effectiveness of mobile-based MOUD treatment and identify strategies for enhancing patient engagement, reducing barriers to care, and improving retention outcomes in comparison to brick-and-mortar OTPs.

This study is part of a multi-method evaluation of CODAC’s MHU. The larger multi-method evaluation includes a chart review of patient health utilization and interviews with patients and stakeholders to characterize the MHU utilization and outcomes. The present study aims to examine the perspectives of stakeholders with the brick-and-mortar and MHU on the experiences of MOUD provision, barriers and facilitators influencing treatment engagement, experiences, perceptions, and beliefs of patients receiving MOUD at MHUs. By addressing these questions, this study contributes to a growing body of research on mobile-based MOUD services, offering insights into patient-centered strategies for optimizing access, retention, and long-term recovery outcomes.

## Methods

To structure our approach, we applied the Exploration, Preparation, Implementation, and Sustainment (EPIS) framework, integrating key principles from the Health Equity Implementation Framework (HEIF) to ensure a comprehensive and equity-focused analysis of the MHU model [11, 12]. These frameworks guided the development of interview protocols, allowing us to explore key factors influencing MHU implementation and service delivery. The interview guides were refined through consultations with key stakeholders and focused on multiple domains, including treatment experiences, barriers and facilitators to engagement and retention, perceived health and social impacts, attitudes toward the MHU, service delivery perceptions, and recommendations for improvement.

Between August and November 2024, we conducted semi-structured qualitative interviews with stakeholders to capture diverse perspectives on MHU implementation and operations. Interviews lasted an average of 54 min (ranging from 38 to 89 min) and were conducted by research staff with expertise in qualitative methods, including a master’s-level program coordinator, a doctoral candidate, and a doctoral-level physician scientist. All interviews were audio-recorded with participants’ informed consent.

To examine the factors influencing MHU delivery across different contextual levels, we recruited 15 stakeholders representing a range of roles. These included clinicians and staff directly involved in MHU operations (*N* = 5; e.g., counselors, peer support specialist), clinicians and staff from CODAC’s brick-and-mortar OTPs (*N* = 5), CODAC leadership and administrators (*N* = 2), staff and/or leadership from a community entity (*N* = 2) and the State Opioid Treatment Authority (*N* = 1). Prior to participation, all stakeholders were informed of the study’s procedures, risks, and benefits, and provided informed consent. Interviews were conducted primarily via virtual platforms. Saturation of themes was achieved after the first 12 interviews, as determined by the interviewers and broader study team; across interviews, concepts repeatedly arose until no new topics were breached. An additional three interviews were conducted for confirmation of saturation until recruitment stopped. Stakeholders who were eligible to accept compensation based on their employer’s guidelines received $50 for their participation.

Interviews were conducted on a virtual conferencing software with artificial intelligence-generated transcripts. Research staff conducted a rigorous review of all transcripts, comparing them against audio recordings to ensure accuracy. Coding and analysis followed a structured, iterative approach. Initially, two coders independently reviewed six transcripts, taking detailed memos and debriefing after each round to refine the interview process. This iterative approach allowed for real-time refinement of interview questions, particularly to probe emerging themes such as housing stability, employment, take-home medication access, and shifts in drug supply. A structured codebook was developed, aligning with key interview domains and incorporating emergent themes. The codebook included domain names, codes, operational definitions, and illustrative quotes. An initial set of six transcripts from different stakeholder groups was double-coded by two researchers, followed by consensus discussions to refine code definitions and resolve discrepancies. Additional inductive codes were incorporated based on emerging themes. Once coder agreement was reached, the remaining transcripts were single-coded, with one researcher analyzing patient interviews and another coding stakeholder interviews.

A template-based thematic analysis was employed, grouping related codes within overarching domains. Throughout the coding process, themes and subthemes were refined iteratively [13]. Upon completing coding, the research team conducted a final review of the thematic structure, ensuring consistency and coherence across categories. To facilitate data organization and synthesis, NVivo software was used for qualitative analysis [14]. This study was approved by the Brown University Institutional Review Board.

## Results

### Themes from stakeholder interviews

**Fig. 1:**
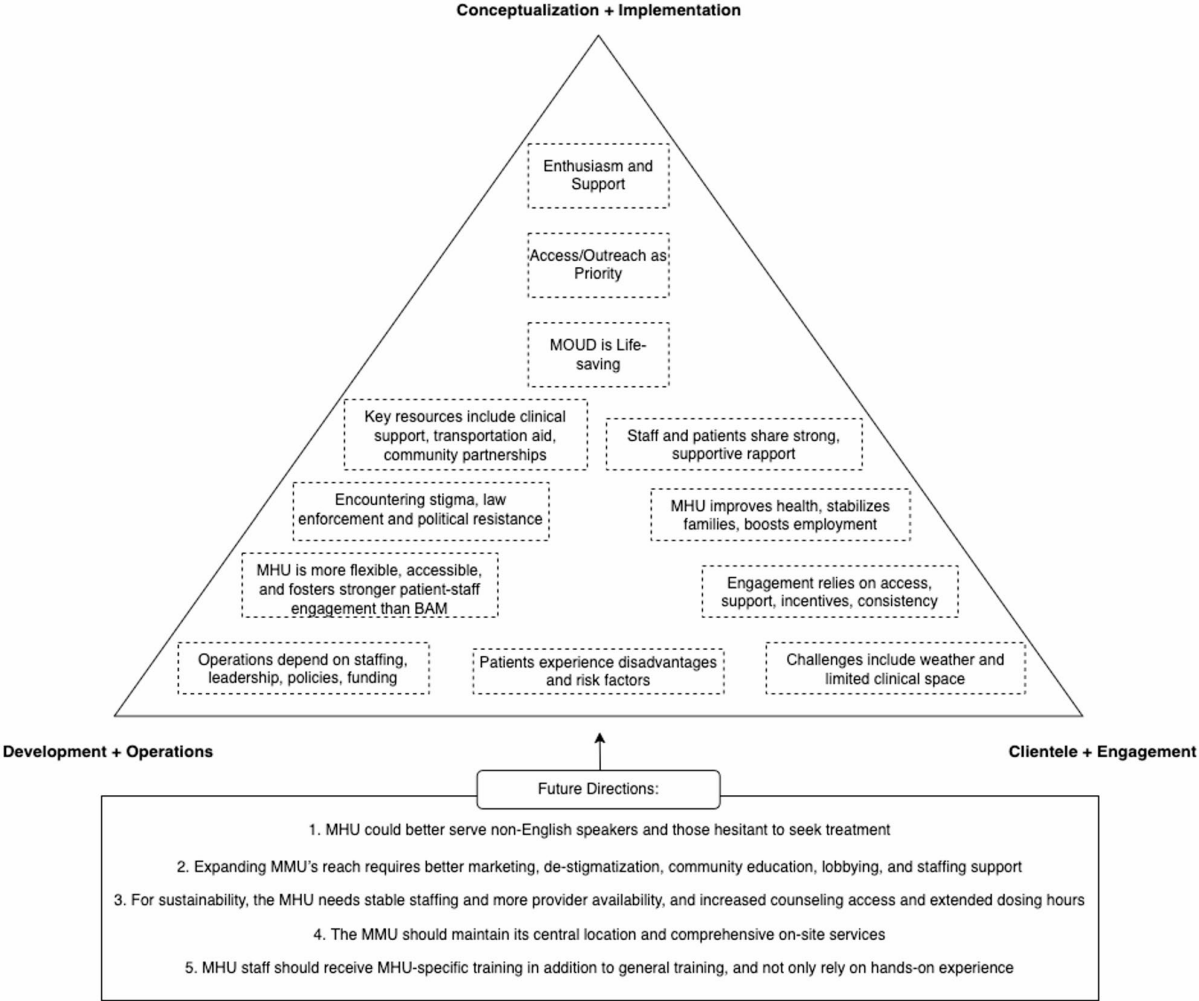
Illustration of codes embedded within the domains. Acronyms used: MHU = Mobile Health Unit. MOUD = Medications for Opioid Use Disorder

Interviews with participants yielded twelve codes within the framework structured by the interview guide. Consistent with the EPIS framework, findings are presented with emphasis on the Implementation and early Sustainment phases. Themes reflect stakeholders’ experiences translating the MHU model into practice, navigating operational and contextual barriers, and sustaining engagement with patients and community partners. Although EPIS informed the overall organization of findings, themes were derived inductively and are presented narratively to reflect stakeholders’ lived experiences and priorities. We present the findings in a narrative structured by the codes. Figure 1 illustrates the codes within three domains of interview questions embedded within the interview guide: (1) Conceptualization and implementation, (2) Development and operations, and (3) Patients and their engagement. We also further discuss recommendations and future directions distilled from our participant responses. Table 1 illustrates the demographic characteristics of the participants.

**Table 1:** Characteristics of interview participants

	*N* = 15	%
Age, years (M; SD)	48.4	13.9
**Gender**		
Female	9	60.0
Male	6	40.0
**Race** (all that apply)		
White	11	73.3
Black or African American	5	33.3
**Hispanic**,** Latino or Spanish origin**	1	6.7
**Educational attainment**		
Did not complete HS	1	6.7
HS or equivalent	0	0.0
Some college	2	13.3
Associate’s degree	1	6.7
Bachelor’s degree	3	20.0
Graduate degree	8	53.3
**Stakeholder type**		
CODAC staff, mobile unit	5	33.3
CODAC staff, non-mobile unit	5	33.3
CODAC leadership	2	13.3
Community entity, staff or leadership	2	13.3
Government	1	6.7

M = mean, SD = standard deviation, HS = high school

Themes within the Implementation phase describe stakeholders’ experiences translating the MHU model into practice, including early enthusiasm, operational workflows, patient engagement strategies, and day-to-day challenges inherent to delivering MOUD in a mobile setting. Subthemes include: (1) Enthusiasm and support for the MHU, (2) Access and outreach as a priority, (3) MOUD as a lifesaving intervention, (4) Flexibility and patient–staff engagement compared to brick-and-mortar clinics, and (5) Early encounters with stigma, law enforcement, and community resistance.

Themes within the Sustainment phase reflect stakeholders’ perspectives on the conditions necessary for maintaining and scaling the MHU over time. These findings emphasize structural, financial, and workforce factors that influence program stability, adaptability, and long-term impact. Subthemes include: (1) Staffing, leadership, policies, and funding as determinants of sustainability, (2) Environmental and infrastructural constraints, (3) Workforce retention and training needs, (4) Community partnerships and resource dependencies, (5) Concerns about long-term funding and policy support.

### MHU Conceptualization and implementation

#### Enthusiasm and support for the MHU

Overall, participants widely endorsed the MHU as a critical intervention that enhanced treatment access and expanded the reach of MOUD services. Participants expressed strong initial enthusiasm for the implementation of the MHU, highlighting its potential to bridge critical gaps in OUD treatment access. Many noted that the MHU addressed logistical and regulatory barriers inherent to MOUD, particularly the requirement for in-person contact to initiate treatment. A participant emphasized the MHU’s unique ability to reach those experiencing housing instability or living in more remote areas:

“I felt that it was a fantastic idea because of the way that methadone-based MOUD care is regulated. It requires in-person contact with patients to initiate treatment, and that can be a real barrier for people who are unhoused or transient… The opioid use disorder crisis in Woonsocket is quite severe… the mobile unit was a great way to approach trying to help, trying to get care out to people who need help out there.” [3012, Brick-and-mortar staff]

According to stakeholder participants, engaging patients from outlying areas was difficult and the MHU facilitated treatment access. A participant, who had previously worked at a brick-and-mortar clinic, described the issue:

“It’s harder to catch those people or have them come in or deliver services to them… So it [the MHU] is beneficial in that sense, like in the rural areas.” [3013, Brick-and-mortar staff]

#### Access/outreach as priority

Participants consistently highlighted the MHU’s role in expanding access to treatment by meeting patients where they are, particularly for underserved populations facing structural barriers to care. Individuals who are unhoused and experiencing economic hardship often encounter significant obstacles to seeking treatment. The MHU was described as a solution that brought services directly to those in need, eliminating key barriers such as transportation costs and stigma associated with attending traditional treatment sites. A participant emphasized:

“The biggest priorities are to meet [patients] where they are, especially the underserved minority populations, [unhoused] populations, people who just don’t have the economics to…even get a bus ticket.” [3004, Community entity/government personnel]

Beyond methadone provision, the MHU was valued as a point of contact for patients to engage with broader health services. Participants underscored that many patients often have additional unmet medical needs, including unmanaged chronic conditions like hypertension and diabetes. A participant described this holistic approach:

“…the population doesn’t keep track with their other medical needs, such as…diabetes, high blood pressure. A lot of those things often get neglected or just not easily accessible. So…the mobile unit is another point of contact to reconnect people to some of these services.” [3007, Brick-and-mortar staff]

### MHU development and operations

#### Operations depend on staffing, leadership, policies, and funding

##### Further sub-theme: Operations depend on staffing, leadership, and human resource policies

 The success and sustainability of the MHU were widely recognized as being contingent on adequate staffing (i.e., sufficient staff needed for shifts, MHU-dedicated staff), strong leadership, effective human resource policies, and stable funding. Participants highlighted the ongoing challenges of hiring and retaining personnel for the MHU.

“Staffing in general is challenging for all medical facilities in the state… It’s challenging to get trained staff. So I do think staffing is a real challenge for any of our sites, but particularly for the mobile unit… Most of the staff aren’t local to the mobile unit, so that can be a little tough…” [3012, Brick-and-mortar staff]

To bridge the gap of staff needed for the MHU, a couple of participants described how counseling and case management services were provided through a rotation of staff from the Providence location rather than a fully dedicated MHU team:

“The only thing that could be somewhat of an obstacle is that they’re not currently fully staffed… Right now, what happens is I essentially loan out counselors from Providence. If they have a day that they can schedule in advance, they go out to the mobile unit to help provide counseling services and case management, just so there is always some form of clinical presence there.” [3008, MHU staff]

##### Further sub-theme: operations depend on funding

Funding was a recurrent concern, with participants highlighting low reimbursement rates from insurance companies as a key limitation. Some noted that increased funding could be used to improve staff retention by offering more competitive salaries and incentives, particularly given the challenges of working in a mobile setting (i.e., smaller spaces, working partially outdoors):

“If we had additional funding, then we could probably give more competitive salaries, which would attract [staff]…” [3011, MHU staff]

and

“Reimbursement rates for insurance companies are always an issue…if those were higher, we would have a “higher” payments from insurance companies which would enable use to run the program more effectively…” [3010, MHU staff]

#### MHU is more flexible, accessible, and fosters stronger patient-staff engagement than brick-and-mortar

Participants emphasized that the MHU provided greater flexibility, accessibility, and opportunities for patient-staff engagement compared to brick-and-mortar treatment facilities. The mobility of the unit was seen as a key advantage, both in terms of its ability to reach patients where they were and in its adaptability to community needs (e.g., provide needs for unhoused patients). A participant highlighted that, unlike a fixed-site clinic, the MHU could relocate if needed, mitigating concerns about community resistance and allowing services to continue uninterrupted:

“The flexibility… even if they said tomorrow, ‘Hey, you can’t be in this parking lot,’ we’d be able to find a different space in the general downtown area… That helps a little bit with NIMBYism, because the fact that it’s not a brick-and-mortar space probably allows a little bit more freedom of movement… There’s, you know, not everybody is a fan of having a methadone clinic right in their backyard, you know. But the bus makes it a little bit… less scary.” [3015, Community entity/government personnel]

Beyond its flexibility, the location of the MHU played a crucial role in reducing transportation barriers, particularly for patients who lived in the immediate community. Participants noted that brick-and-mortar facilities often required long commutes, which could be prohibitive for individuals with limited financial resources or unreliable transportation. A participant explained how the MHU’s proximity within the neighborhood significantly enhanced treatment access:

“For our patients, based on the location, it’s more accessible… If they had to go someplace else, then I think our next closest location is like 15 miles away. So that’s 30 minutes. So we take a minimum of an hour if they had transportation-that’s a long way. It costs. You know, the gas and the wear and tear on someone’s car. So having the unit in the neighborhood fully accessible is a big impact.” [3011, MHU staff]

Beyond logistical advantages, participants highlighted that the MHU fostered deeper relationships between patients and staff, creating an engaged and supportive environment compared to traditional clinics. A participant observed how this environment contributed to a greater sense of community and support:

“In the brick-and-mortar clinics, people just want to get in, get their medication, and get out. But with the mobile unit, it’s a little bit different because there’s people there, and you get used to seeing the same person every day. And they know your name-not just one person, but all of them… There’s more engagement with the peers and staff, just due to the way the unit is set up. I think those relationships sometimes build a little quicker in the mobile unit than they would in the brick-and-mortar.” [3009, Leadership]

#### Encountering resistance: stigma, law enforcement, and politics

The implementation and operation of the MHU have been shaped by persistent challenges related to stigma, law enforcement presence, and political resistance. At other treatment clinic locations, law enforcement activity near treatment facilities created significant barriers for patients, often forcing individuals’ decisions as they weighed the risk of legal consequences against their need for life-saving treatment. A participant explained:

“There would be instances where [name of another city in the state] police… would do traffic stops in the neighborhood around our building, knowing that people would be coming to our facility for services. If there was somebody that may have had a warrant, and law enforcement being familiar with that individual, they might happen to be in the area when they would be coming to access care… So people had to leverage: ‘Do I go get my medication today, or is it worth the risk of possibly getting picked up on a bench warrant?’” [3005, Brick-and-mortar staff]

However, proactive and continued collaboration with law enforcement emerged as a key strategy to reduce barriers to care. Some MHU staff proactively engaged local police departments to educate officers on the importance of OUD treatment, ultimately fostering more supportive relationships. These relationships were fostered by prior, long-standing relationships between the organization and law enforcement. In some cases, law enforcement officers began referring individuals to the MHU instead of arresting them, demonstrating a shift toward harm reduction. Breaking down misconceptions between treatment providers and law enforcement led to mutual understanding and improved outcomes:

“I was told many times before that treatment services cannot have a relationship with law enforcement. And I flipped it upside down… The police were saying, ‘We don’t want to arrest this person. Can we bring them to you?’… And so it took a while to realize that our community can work well with law enforcement… We just let them know we’re here and nudge people our way, and that works.” [3009, Leadership]

Beyond law enforcement challenges, the MHU also faced political resistance, particularly among more conservative local officials who viewed harm reduction services with skepticism. Some city officials actively sought to prevent the MHU from operating by citing local ordinances and zoning regulations. In one isolated case, legal action was necessary to counter efforts by city leadership to shut down services:

“When [community partner] first invited CODAC to come to [the community partner’s] parking lot, they were there for about six months, and then the mayor and her administration attempted to say that we were breaking some ordinances… CODAC engaged their attorneys, and [the community partner] engaged…attorneys… and they kind of backed off. But we were never surprised when politics reared its ugly head.” [3014, Community entity/government personnel]

Fortunately, MHU staff were not personally required to respond to this incident. Opposition to the MHU often reflected broader community stigma toward individuals with substance use disorders. Some local narratives framed OUD treatment services as attracting unhoused individuals and people with criminal backgrounds, rather than recognizing these services as critical public health interventions. Over time, however, continued operation of the MHU helped shift community perceptions, as residents saw that the unit did not create the negative impacts they feared:

“The longer the unit is there, the more people get used to it. They see that it’s not causing anything adverse… But the narrative in Woonsocket is still that, if it weren’t for human service organizations, we wouldn’t be attracting all these low-income and unhoused people-without considering the fact that they’ve been here for generations.” [3014, Community entity/government personnel]

#### Key resources include clinical logistical support, transportation aid, and community partnerships

Participants highlighted the critical role of resources and external partnerships in supporting both patients and staff within the MHU. Access to clinical logistical support (e.g., harm reduction supplies), transportation assistance and community services was identified as essential to the success of the unit. Practical resources such as transportation assistance were critical for patients accessing care. Many individuals receiving services at the MHU lacked stable transportation, making bus passes and other transit options necessary to ensure continuity of care.

“A lot of times I have to apply for a bus pass for them. A lot of them don’t have adequate transportation.” [3006, MHU staff]

In addition to transportation, harm reduction resources (e.g., clean needle and wound care kits) were provided through partnerships with local community agencies. While participants viewed these partnerships as beneficial, they identified persistent gaps in meeting broader social needs, particularly related to food security, clothing, and deeper collaboration with local service providers:

“There have been a couple of community agencies that have helped provide us with clean needle kits, clean wound kits that have been very helpful… I think there are some areas we could do better in-reaching out and getting more resources in the area, like food pantries, meeting the clothing needs of patients, and aligning more with other community agencies. Not just providing a list of what they are, but actually collaborating in person. That would be really beneficial.” [3010, Leadership]

### MHU patients and their engagement

Please note that no patients were included in this study and that all findings reflect participant perceptions rather than direct patient-reported experiences.

#### Patients experience disadvantages and risk factors

Participants described the complex and multifaceted challenges faced by patients receiving care at the MHU, including housing, unemployment, mental health issues, high rates of trauma, and limited access to transportation. A significant portion of the patient population was unhoused, which exacerbated their vulnerability to violence, victimization, and financial instability. A participant emphasized the profound disadvantages that many patients experience, noting:

“A large percentage are chronically unhoused. A large percentage [have disabilities], unemployed. Significant, significant trauma history from many, if not all, high [Adverse Childhood Experience] scores.” [3012, Brick-and-mortar staff]

These vulnerabilities also increase exposure to harm, making engagement in treatment even more critical for survival. Participants stressed that despite these risks, patients who attended the clinic were actively making efforts to improve their health:

“The large majority of the population is unhoused], which comes with a variety of variables - being at risk for being robbed, being victimized… But when they’re at the clinic, they’re at least making efforts to take better care of themselves.” [3011, MHU staff]

While many of these factors contribute to instability and high-risk behaviors, participants also emphasized the role of the MHU in fostering a welcoming and nonjudgmental environment for patients who might otherwise disengage from care. The sense of community within the MHU encouraged continued engagement, even perhaps among individuals actively using substances:

“It does work well for those high-risk, high-acuity people… It’s that family-oriented environment. Everybody is welcoming, so they feel more comfortable showing up. And even if they’re not in their best state, maybe really high on cocaine or something, they still show up. Because they know these people are kind to them on a day-to-day basis, and they won’t judge them.” [3003, Brick-and-mortar staff]

#### Challenges include weather and limited clinical space

While the MHU provides critical, life-saving services, its operation is not without significant challenges. Participants highlighted unpredictable weather conditions, limited clinical space, staff retention issues, and inconsistent funding as major barriers to sustaining and expanding care. Since implementation, adaptations to unit services have resulted from these challenges. For weather-related issues, the unit expanded take-home dose(s) provision in anticipation of inclement weather:

“You would think, oh, it’s heavy. It probably goes through the snow, but it was just horrible. We had to get there-they needed their doses… I pulled up, and I actually got there before the mobile unit, and I felt so bad. There were like four patients out there freezing cold in the snow, but we’re not allowed to let them sit in our car for heat. So I had to call my boss… We had to change the policy to give them a take-home bottle whenever snow was forecasted.” [3001, MHU staff]

and,

“Weather. Yes, you know, you have the elements. You have things that can get in the way when it comes to weather issues. You’re not in one solid place. So you can have environmental issues that can play a role in functioning as a unit.” [3010, Leadership]

Limited clinical space was another frequently cited challenge. Unlike brick-and-mortar clinics, the MHU has only one enclosed office space available for private patient interactions, forcing providers to adapt to open environments with makeshift dividers and sound machines. The lack of dedicated office space made it difficult for some clinical staff, especially those accustomed to traditional settings, to adjust to the MHU’s outreach-based model:

“It’s not a very clinically based environment in the sense that there’s not office space. There’s like one office on one of the buses. The other one has a divider and a sound machine… A lot of people in a social work setting like offices. So, you really have to be a professional who wants an outreach job.” [3003, Brick-and-mortar staff]

#### MOUD is lifesaving

Stakeholder participants emphasized the life-saving role of MOUD, describing its ability to stabilize patients, reduce harm, and support long-term recovery. Stakeholders had extensive professional, and some personal, experience with MOUD at the MHU and other settings. Across interviews, participants highlighted the profound impact of methadone and buprenorphine in helping individuals regain control over their substance use and, in many cases, their broader lives. A participant described how patients receiving MOUD develop a greater sense of self-awareness and responsibility regarding their substance use:

“I have seen my patients, with them being on whether it’s methadone or Suboxone, grow more cognizant of what they take. They grow more cognizant of, ‘I may still struggle with some type of substance use, but I haven’t used in three days or I use less.’ So they have more of a growing responsibility to stabilize themselves.” [3002, MHU staff]

And,

“It allows them to function. They’re not at risk for… being in withdrawal and possibly, you know, leading to death. It allows a person to…function better in their life. It makes different components of their life-family, work, self-esteem, parenting-better.” [3011, MHU staff]

#### Engagement relies on access, support, incentives, and consistency

Participants described that sustained patient engagement in MOUD services depends on ease of access, supportive relationships, meaningful incentives, and consistency in care delivery. Participants highlighted that removing logistical barriers was critical to ensuring that individuals could initiate and continue treatment. The MHU prioritized low-barrier entry, allowing patients to walk in without an appointment, receive immediate assessments, and, when necessary, access transportation support to connect with other services:

“You can show up at the unit at any time between 6 AM and 10 AM and be able to access services… If we can find any type of documentation as to who the patient is, then we don’t allow ID or insurance to be a barrier. Once the patient accesses CODAC, we’ll figure out how to get them intaked, assessed, and dosed that day.” [3011, MHU staff]

Beyond access, supportive relationships with staff and peers were key to keeping patients engaged, according to participants.

“I let them know that we work as a team. So if they need something, I will collaborate with any other staff to help them get what they need… Even if it’s something we’re not able to provide, they can feel at ease to reach out to myself or any other staff.” [3002, MHU staff]

Incentives also played a major role in fostering engagement, particularly in the early stages of treatment. Soon after the MHU was implemented, small monetary incentives were provided to participants who attended the unit. Over time, as patients built relationships with staff and stabilized in their recovery, they became intrinsically motivated to seek care:

“When we first started out, we would give them little $10, $15, $20 Walmart gift cards. And they would break down the door. Of course, they’re [unhoused]-that’s food… But after about six months, we didn’t even have to. They would just come in on their own and say, ‘I need to get [help with social services]’ or ‘I need food assistance.’” [3001, MHU staff]

Another powerful motivator for engagement was the ability to earn take-home doses of methadone (i.e., earning the ability to take one’s daily dose of methadone at home rather than the unit), reducing the frequency of clinic visits and offering a sense of autonomy. Participants reported that patients were highly invested in meeting treatment goals that would allow them to increase their take-home supply, making this one of the strongest retention tools that stakeholders saw:

“That’s arguably one of the largest external motivators, especially in early recovery-‘Okay, what do I have to do to earn more take-homes?’ And that gets patient buy-in to engage initially. Then, as people get more established in their recovery, you naturally see that external motivation translate into intrinsic motivation.” [3005, Brick-and-mortar staff]

Finally, consistency in care delivery and staffing was essential to long-term engagement. Patients valued staff members who followed through on commitments, developed therapeutic relationships, and provided reliable, nonjudgmental support:

“Having worked with people for years and years, consistency-saying you’re going to do something and actually doing it-is huge… Once a patient develops a rapport with a counselor and knows they’re here to help them, that therapeutic relationship is key to keeping people engaged.” [3013, Brick-and-mortar staff]

#### MHU improves health, stabilizes families, and boosts employment

Participants who worked at the MHU described how the MHU plays a transformative role in improving individual health outcomes, stabilizing families, and supporting patients in rebuilding their lives. Given that the MHU provided direct, low-threshold access to MOUD (specifically methadone), participants often described the benefits of the MHU as synonymous with benefits of methadone. As a participant described, methadone offers patients “breathing room” to reassess their lives and begin working toward stability:

“Using drugs is the chase game. You got to find it. You gotta go get it. It’s a whole process. So medications like methadone give people some breathing room to think about what they really want out of life, without having to go through the pain, the physical discomfort of withdrawal.” [3006, MHU staff]

Beyond addiction treatment, the MHU provides a range of essential health services. These services (conducted on days that are separate from dose clinics) are particularly crucial for unhoused patients who may otherwise lack access to routine healthcare. A participant shared the range of services provided by the unit:

“There is a nurse there who does blood pressure checks, wound care, and just makes sure that everything is okay before referring them to a primary care doctor… That has been super helpful, and that is happening during mobile unit outreaches.” [3013, Brick-and-mortar staff]

Participants also underscored the profound impact of MOUD on family stability, particularly for patients who are parents or caregivers. By reducing the time and energy spent on seeking substances, MOUD allows individuals to refocus on their children, partners, and extended family members. In many cases, stakeholders reported that patients who stabilize on treatment bring their loved ones into care, breaking cycles of generational trauma and addiction. Brick-and-mortar and MHU staff described the benefits of involving families in MOUD treatment:

“A lot of our patients are parents. It stabilizes their ability to care for their children or their caregivers for their parents… Oftentimes, our patients’ parents also have use disorders. Their children also have use disorders. If we can establish contact, they’ll bring their parents in. They’ll bring their children, their partners. Then we can care for all of them.” [3012, Brick-and-mortar staff]

And,

“I see families more invested. The ones that do have family involvement - I see their families come and drop them off, come with them. So, I do see more family involvement with those who still have family in their lives.” [3002, MHU staff]

Beyond family stability, MOUD significantly improves long-term social and economic outcomes, with many participants reporting that patients transitioned from being unhoused, unemployed, and having legal involvement to stable employment and independent living. A participant, reflecting on over a decade of experience working with patients, described witnessing countless transformations from rock bottom to full-time employment and family stability:

“I’ve been working for CODAC for 14 years, and I’ve seen people turn their lives completely around. You know, from the ground up, hitting rock bottom. And then, you know, now being productive in society, having full-time jobs, families, not having legal involvement anymore… Doing a lot of really positive, inspirational things.” [3008, MHU staff]

#### Staff and patients share strong, supportive rapport

Staff shared that most patients often described staff as approachable, familiar, and committed to fostering a comfortable and trusting environment. While staffing challenges were noted, particularly regarding turnover and the absence of a permanent counselor on-site, participants emphasized that the core team members present on a regular basis had developed deep and meaningful relationships with patients. A participant highlighted how a peer specialist had become a familiar and trusted presence:

“There’s one person that’s been there on a regular basis, who is a peer specialist, and when I’ve been there, he’s always had a good rapport with the [patients]. People know him. They feel comfortable with him. Even with the doctor, I always felt like there’s been a good rapport between the doctor and the patient.” [3007, Brick-and-mortar staff]

Participants noted that the collaborative atmosphere among staff contributed to positive patient experiences, as staff members worked together to create a sense of familiarity and consistency. Familiarity and consistency were fostered through having the same staff working overtime, small talk with individuals and groups of patients, and offering services routinely (i.e., doctors always on Fridays). A participant reflected on the strong relationships built over time, emphasizing that the vast majority of patients engaged respectfully and productively with staff:

“We all had started together, and we all just built this great work relationship and a great rapport with the patients there… Out of the year that I worked there, when I left, there were 89 patients on the mobile unit. And out of that, I can honestly say I probably had to do maybe five behavior contracts [e.g., contract created with staff when patients exhibit challenging behaviors on site].” [3001, MHU staff]

The following table (Table 2) summarizes the barriers and facilitators discussed above.

**Table 2:** Summary of identified barriers and facilitators by subthemes

Barriers	Facilitators
Operations depend on staffing, leadership, policies, and funding	Enthusiasm and Support for the MHU
Encountering resistance: stigma, law enforcement, and politics	Access/outreach as priority
Patients experience disadvantages and risk factors	MOUD is lifesaving
Challenges include weather, and limited clinical space	MHU is more flexible, accessible, and fosters stronger patient-staff engagement than brick-and-mortar
	Key resources include clinical support, transportation aid, and community partnership
	Engagement relies on access, support, incentives, and consistency
	MHU improves health, stabilizes families, and boosts employment
	Staff and patients share strong, supportive rapport

Note. MHU = Mobile Health Unit. MOUD = Medications for Opioid Use Disorder

### Summary of future directions as discussed by participants

Participants identified several key areas for improvement and expansion to enhance the MHU’s reach, effectiveness, and sustainability. Future directions included improving marketing and outreach, de-stigmatization efforts, increasing community education, securing long-term funding, and addressing staffing shortages. Additionally, participants highlighted the need to improve services for underserved populations, such as unhoused individuals, non-English speakers, and those hesitant to engage in treatment.

#### Expanding outreach and marketing

Many participants emphasized that better marketing and public awareness efforts could significantly increase patient engagement. Current outreach efforts (e.g., flyers at other OTP sites, passive word-of-mouth) were seen as insufficient, with participants suggesting greater use of social media, flyers, and ad placements to ensure that more people know about the MHU’s services:

“I think marketing is something that we really could probably improve upon… Posting flyers in a community center, a local soup kitchen, or places where people frequent… Also, social media is an area in which we’re probably lacking in presence. Even just one or two ad placements could boost visibility.” [3005, Brick-and-mortar staff]

#### De-stigmatization and community education

Stigma arose as a significant factor when discussing primary barriers to MHU utilization and growth. Participants expressed the need for a large-scale de-stigmatization campaign to change public perceptions of MOUD. A suggested approach was educational initiatives in schools, workplaces, and community settings, particularly targeting industries with high rates of substance use, such as construction and labor-intensive jobs:

“A campaign to de-stigmatize [MOUD] services would be a really great thing… Community education in schools, hospitals, and large businesses like Amazon or Textile. If we can destigmatize this-if a person needed insulin, no one would say anything. Addiction should be treated the same way.” [3011, MHU staff]

#### Improving access for underserved populations

Participants noted several barriers preventing key populations from accessing services, including unhoused individuals who need housing support, non-English speakers who face language barriers, and those hesitant to engage due to stigma or lack of perceived benefit. Addressing these concerns would require better community partnerships, targeted outreach, and improved language accessibility:

“We do serve a lot of people from other cultures, but we don’t always have staff who can address the language barrier. That’s one area to look into.” [3007, Brick-and-mortar staff]

”I wish we could do more for the [unhoused] population… But all we can do is referrals. If they’re [unhoused], they [may] have a criminal record, and that makes it harder for them to access public housing.” [3001, MHU staff]

#### Enhancing staffing and service delivery

Participants recommended hiring additional providers, increasing doctor availability, improving space management for intakes, and expanding counseling services:

“It would be ideal to have a second nurse. At other CODAC sites, they have more than one nurse, which helps when there’s an issue… But I know that’s unrealistic for a mobile unit.” [3002, MHU staff]

#### Maintaining a central location and expanding services

Participants valued the MHU’s location on a main road near bus routes and shelters, making it easily visible and accessible to the target population. Maintaining this strategic placement was seen as crucial for continued engagement:

“The mobile unit is on a main road. It’s very easy to see that there’s treatment there. That’s where the [unhoused] population frequents, and they see it’s available.” [3003, Brick-and-mortar staff]

Additionally, participants hoped for expanded services, including longer dosing hours, a broader array of medical care, and harm reduction tools like community drug checking:

“I hope the unit can provide a broader array of services-not just methadone and Suboxone, but basic medical care… And more coordination with primary care and behavioral health providers.” [3014, Community entity/government personnel]

”A lot of people are polysubstance users, not just using opioids. It would be good to provide education and drug checking-something where people can say, ‘I bought this bag, it looks funny, can you test it?’ That kind of ground-level service would be very effective.” [3015, Community entity/government personnel].

#### Improving training for MHU-specific needs

While CODAC staff receive general training, participants noted a lack of specialized training for the MHU environment. Instead, most new staff members learn on the job, which could be improved by developing structured MHU-specific training:

“All our staff are well-trained in motivational interviewing, but there’s no firm training specific to the [MHU]. It’s more of an internal, hands-on training from former staff.” [3009, Leadership]

## Discussion

This study explored the implementation, impact, and challenges of a MHU providing MOUD from the lens of critical stakeholders inclusive of unit staff and administrators, staff and administrators from brick-and-mortar clinics in the same network, and other invested community partners. Participants widely endorsed the MHU as a critical intervention for expanding treatment access, particularly for individuals facing significant barriers such as housing and economic instability, and limited transportation. The MHU’s flexibility and ability to meet patients where they are was seen as a major strength, reducing logistical obstacles and fostering strong patient-staff relationships. Beyond methadone dispensing, the unit provided essential services, including harm reduction, medical screenings, and social support, helping patients stabilize their lives. However, several operational challenges were identified, including staffing shortages, inconsistent funding, limited clinical space, and difficulties retaining personnel willing to work in an outreach-based environment. Participants also noted the influence of external factors such as stigma and political resistance, which at times deterred patients from seeking care. Despite these barriers, the MHU was credited with improving patient engagement, reducing illicit drug use, and promoting family stability and employment.

Of note, the MHU was widely endorsed by participants as a crucial intervention to reduce logistical and structural barriers to MOUD. By providing services directly to patients in their communities, the MHU eliminates the need for transportation, reduces stigma, and facilitates engagement for those who might otherwise struggle to access care [5]. Patients experiencing lack of housing and economic hardship particularly benefited, as they often face significant financial and logistical obstacles to attending brick-and-mortar clinics [15, 16]. Indeed, participants were supportive of expanding the MHU model for more communities to benefit, though barriers, such as the key concern of funding limitations, would need to be addressed. Participants noted that low insurance reimbursement rates and the precarious nature of grant-funded projects threatened the sustainability of the MHU. While CODAC has been able to maintain funding thus far, there was broad concern that support could taper off, leading to reduced services or program termination. Securing long-term, stable funding sources will be essential to ensuring the continued operation and expansion of mobile MOUD services.

Beyond medication provision, participants emphasized the broader health and social benefits of the MHU. Many patients entered care with unmanaged chronic conditions such as hypertension and diabetes, and the MHU provided an opportunity to reconnect them to primary care services. The ability of the MHU to integrate medical, social, and harm reduction services underscores its importance as a comprehensive public health intervention. This is important in aligning with the principle of integrated care models for people with SUD. Studies on integrated care models suggest that combining MOUD with primary care services leads to better overall health outcomes, improved retention in treatment, and increased patient engagement [17–19]. The MHU’s role in reconnecting patients to primary care services mirrors successful strategies seen in low-barrier treatment models, which emphasize the integration of addiction treatment, preventive care, and social support [18]. 

Participants emphasized that MOUD is lifesaving, reducing the risk of overdose and enabling patients to regain control over their lives. Many reported that patients developed greater self-awareness and responsibility, demonstrating improved engagement in their treatment and personal well-being. Additionally, participants noted the profound impact of MOUD on families, as treatment stability often allowed patients to rebuild relationships with children, partners, and extended family members, highlighting the intergenerational impact of addiction treatment. Studies show that parental substance use disorder is strongly associated with adverse childhood experiences (ACEs) and family dysfunction, but MOUD programs can break cycles of trauma by improving parenting capacity and family engagement [20, 21]. Research on MOUD and family dynamics has found that treatment not only helps patients re-establish relationships with their children and partners but also leads to increased family support, improved social reintegration, and reduced child welfare involvement [22, 23]. Moreover, economic and legal stability were noted as significant benefits of MOUD treatment. Many patients were able to transition to employment, secure stable housing, and potentially avoid legal entanglements. These findings align with broader public health research demonstrating the positive ripple effects of addiction treatment on individuals, families, and communities [24]. 

Environmental factors such as inclement weather and limited clinical space posed additional challenges. Unlike brick-and-mortar clinics, the MHU must contend with snowstorms, extreme temperatures, and other environmental disruptions, which can delay treatment or reduce patient attendance. Limited space within the unit also constrained counseling and intake services, necessitating creative scheduling and workflow adaptations. Specific staff retention strategies in the context of MHU should be explored with the population in future research.

Despite the public health benefits of the MHU, its implementation was potentially met with stigma, political opposition, and law enforcement challenges. In some locations, police activity near treatment sites were perceived to potentially raise uneasiness among justice-involved populations. However, participants reported that proactive engagement with law enforcement helped foster understanding, and some officers even began referring individuals to treatment instead of pursuing punitive measures. These findings suggest that building relationships with law enforcement agencies can reduce harm and improve community trust. Political resistance also emerged as a barrier, with some municipal officials attempting to block MHU operations due to concerns about attracting unhoused individuals or increasing crime. However, participants noted that over time, continued operation of the unit helped shift community perceptions, as residents recognized that the MHU did not contribute to neighborhood decline but instead provided essential services to vulnerable populations. Future efforts to expand mobile MOUD services should include strategic engagement with local policymakers to address misconceptions and advocate for evidence-based harm reduction policies.

A key limitation of this study is the absence of direct patient perspectives; as such, findings related to patient experiences reflect provider and staff interpretations rather than patients’ own voices.

### Future directions for the MHU

While the MHU was seen as a critical resource, its success was hindered by several operational barriers, including staffing shortages, funding constraints, and environmental factors. Participants identified staff retention as a significant challenge, with many providers reluctant to work in the mobile setting due to commuting difficulties, lack of dedicated clinical spaces, and perceived job instability. Studies on MOUD delivery models suggest that high staff turnover is prevalent, and fragmented provider schedules negatively impact patient trust, adherence, and treatment outcomes [17, 25]. Patients are more likely to disengage from care when they experience frequent provider changes or receive treatment from staff unfamiliar with their clinical and social history [26]. 

To address these challenges, participants suggested several future directions, including enhanced marketing and outreach to increase awareness of MHU services, expanded staffing capacity (e.g., additional nursing and provider support), and streamlined intake processes. Participants also recommended expanding services to include harm reduction tools, community drug-checking, and broader medical care to strengthen the MHU’s role as a comprehensive public health intervention. We note that, at the time of writing, CODAC’s MHU increased provider reimbursement rates in an effort to improve retention, though continued evaluation of staffing stability is warranted.

Finally, participants emphasized the importance of long-term sustainability through stable funding, policy advocacy, and strengthened partnerships with housing services, food pantries, and community-based organizations to better address patients’ broader social needs.

## Conclusions

This study highlights the critical role of the MHU in expanding access to MOUD, reducing barriers to care, and improving patient outcomes. While the unit has been widely embraced by patients and providers, ongoing challenges related to staffing, funding, and operational sustainability must be addressed to ensure its long-term success. Future efforts should focus on strengthening community engagement, securing sustainable funding, expanding services, and reducing stigma to enhance the impact and longevity of mobile MOUD programs.

## Data Availability

No datasets were generated or analysed during the current study.
